# Comparison of Antimicrobial Activity of Child Formula Dentifrices at different Concentrations: An *in vitro* Study

**DOI:** 10.5005/jp-journals-10005-1422

**Published:** 2017-06-01

**Authors:** Ritika Malhotra, Shilpy Singla, ND Shashikiran

**Affiliations:** 1 Student, Department of Pedodontics and Preventive Dentistry, People’s College of Dental Sciences & Research Centre, Bhopal, Madhya Pradesh, India; 2 Reader, Department of Pedodontics and Preventive Dentistry, People’s College of Dental Sciences & Research Centre, Bhopal, Madhya Pradesh, India; 3 Head, Department of Pedodontics and Preventive Dentistry, People’s College of Dental Sciences & Research Centre, Bhopal, Madhya Pradesh, India

**Keywords:** Antimicrobial activity, Dentifrices, *Streptococcus mutans.*

## Abstract

**Aim:**

The aim of the present *in vitro* study is to evaluate and compare antimicrobial efficacy of commercially available child’s dental formulas in reduced concentrations containing different forms of fluoride against *Streptococcus mutans* activity.

**Materials and methods:**

The selected dentifrices were prepared in dilutions of 1:1, 1:2, 1:4, 1:8, and 1:16 using sterile pyrogen-free distilled water. Various dilutions of the selected toothpaste slurries were incubated in the agar plate containing pure strains of S. *mutans,* and antimicrobial activity of each was assessed by measuring the diameter of zones of inhibition (in mm). Agar well plate diffusion method and minimum inhibitory concentration (MIC) determination were the methods used in this study. The inhibitory circle of each dentifrice was measured and MIC was achieved by considering the value of diameter of the circle.

**Results:**

The results of the study showed that even at a lower concentration of fluoride, inhibition halos were obtained for all the formulations at different dilutions.

**Conclusion:**

The kid’s formulations having lower fluoride concentration show antimicrobial activity even after dilutions. Thus, commercially, the fluoride concentrations can be further lowered down in the dentifrices, thereby reducing the risk associated with fluoride.

**How to cite this article:**

Malhotra R, Singla S, Shashikiran ND. Comparison ofAntimicrobial Activity of Child Formula Dentifrices at different Concentrations: An *in vitro* Study. Int J Clin Pediatr Dent 2017;10(2):131-135.

## INTRODUCTION

The paradigm shift in the field of dentistry has led to the adoption of specific preventive treatment protocols or intervention, based on the current assessment of caries and/or the level of associated risk factors. There are various preventive protocols practiced today for caries prevention. Fluoridated formulations have been credited to be effectively used in reducing the risk of dental caries and also reversing enamel demineralization.^1^

Levy^2^ has stated that a dentifrice is the most common source of topical fluoride for young children. But they are considered to ingest enough of fluoride from a dentifrice alone to be at risk of conditions like dental fluorosis.^3^ Deliberate ingestion of toothpaste may also occur because of an uncontrolled swallowing reflex of children aged less than 6 years. Ingestion of the fluoridated toothpaste can be controlled by two ways: by reducing the amount of toothpaste dispensed on the toothbrush and by reducing the concentration of fluoride in kids’ dentifrices.^4^ For this reason, special low-F toothpastes (250-500 ppm F concentration) are used for kids. Consequently, the antimicrobial efficacy of low-fluoride toothpastes still remains unclear.

Toothpastes contain active ingredients or additives that perform specific functions, out of which fluoride is the major active ingredient.^5^ Various forms available to deliver fluoride in a dentifrice are sodium fluoride (NaF), sodium monofluorophosphate (MFP), stannous fluoride, amine fluoride (AmF), and combinations thereof. As an alternative to fluoride therapy, herbal formulations and calcium phosphate formulations have also been introduced in kid’s dentifrices.

The primary objective of any preventive therapy using fluoride for children under age 6 years is to achieve the maximum anticarious benefits with the minimal risk of fluorosis.^3^ While many toothpastes claim to have an antimicrobial activity, no research has been done to prove these claims on child’s dental formulations. This study therefore, seeks to evaluate the antimicrobial effect of commercially available child’s dentifrices containing various forms of fluoride and a fluoride-free calcium phosphate formulation, at different concentrations against *Streptococcus mutans* activity.

## MATERIALS AND METHODS

### Dentifrices used

Dentifrices and their fluoride type, concentration, and sugar substitutes selected for the study are as follows:

*Sample A:* Contained 500 ppm of 0.24% NaF

*Sample B:* Contained 500 ppm of 0.38% sodium MFP and xylitol as sugar substitute

*Sample C:* Contained 500 ppm of 0.38% sodium MFP and sorbitol as sugar substitute

*Sample D:* Contained 458 ppm of 0.35% sodium MFP and xylitol as sugar substitute

*Sample E:* Contained 458 ppm of 0.35% sodium MFP and sorbitol as sugar substitute

*Sample F:* Contained 500 ppm of AmF

*Sample G:* Contained calcium phosphate as active ingredient

### Dentifrice Slurry Preparation

The dentifrice slurry was prepared by mixing the calculated amount of toothpastes (10.0 gm) in measured volume (10 mL) of sterile distilled water to give a 1:1 (toothpaste:distilled water) dilution. Further serial dilutions of the slurry were done using sterile distilled water, and four different dilutions of 1:2, 1:4, 1:8, and 1:16 were made.

### Antimicrobial Assay

The prepared dentifrice slurries were evaluated for antimicrobial activity against *S. mutans.* The bacterial suspension was prepared in sterile brain heart infusion broth at 37°C for 24 hours until a turbidity of 0.5 on the McFarland scale was obtained. For each reading, 100 μL of the bacterial suspension was spread evenly on Mueller-Hinton agar plates using sterile cotton swabs.

Modified agar well plate diffusion method was used to evaluate the antimicrobial activity of dentifrice slurry at different concentrations against the test organism. The plates were allowed to dry. After an hour, a sterile 5 mm cork-borer was used to punch five wells (to receive 1:1, 1:2, 1:4, 1:8, and 1:16 dilutions) at equidistance in each of the plates. Using a micropipette, 20 μL of the prepared dentifrice dilutions was introduced into each of the five wells. The plates were then incubated for 48 hours at 37°C.

Zones of microbial inhibition were recorded in millimeter using a digital caliper. The greatest distance between two points at the outer limit of inhibition halo was measured, and was repeated three times. The mean of these readings was documented for each well.

**Table 1:** **Table 1:** Mean value ± standard deviation of zone of inhibition of different samples (in mm) against S. *mutans*

*Zones of inhibition (in mm) of samples*		*Mean values ± Std. deviation*							
		*1:1 dilution*		*1:2 dilution*		*1:4 dilution*		*1:8 dilution*	
A		18.83 ± 1.041*		13.33 ± 0.577*		0.00 ± 0.000		0.00 ± 0.000	
B		15.17 ± 0.289		11.67 ± 0.764**		0.00 ± 0.000		0.00 ± 0.000	
C		8.73 ± 0.115*		0.00 ± 0.000		0.00 ± 0.000		0.00 ± 0.000	
D		22.83 ± 0.289		22.67 ± 0.289		18.50 ± 0.500*		0.00 ± 0.000	
E		29.17 ± 0.764**		19.83 ± 0.289*		11.67 ± 0.764		0.00 ± 0.000	
F		24.60 ± 0.361*		22.00 ± 0.500*		16.07 ± 0.115*		10.97 ± 0.252*	
G		22.33 ± 0.764*		14.67 ± 0.289**		0.00 ± 0.000		0.00 ± 0.000	

A to G: Toothpaste sample; n = 3; *p < 0.05;** p < 0.01

### Statistical Analysis

All data were processed using Statistical Package for the Social Sciences version 10.0 software package (SPSS Inc., Chicago Illinois, USA), and were analyzed statistically using Tukey’s honest significant difference *post hoc* test and analysis of variance test (Table 1). The level of significance was set to be at 5% value.

## RESULTS

**Fig. 1:**
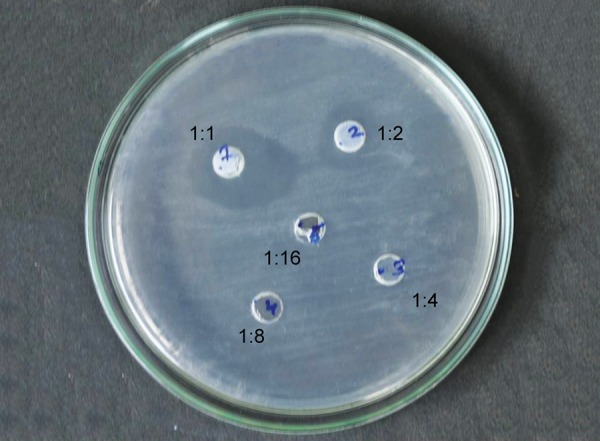
Inhibitory halo for sample A at 1:1, 1:2, 1:4, 1:8, and 1:16 dilutions

**Figs 2A and B:**
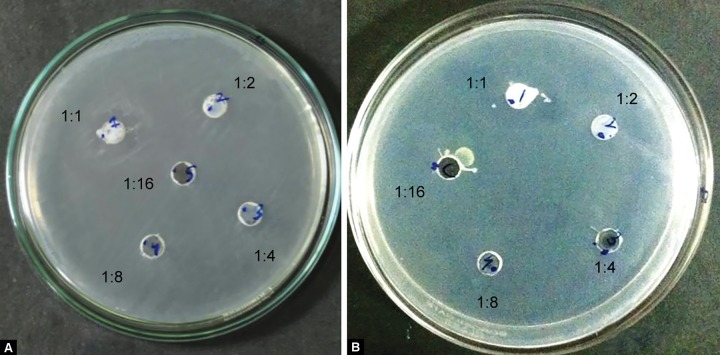
Inhibitory halo for samples B and C (0.38% MFP) at 1:1, 1:2, 1:4, 1:8, and 1:16 dilutions

**Figs 3A and B:**
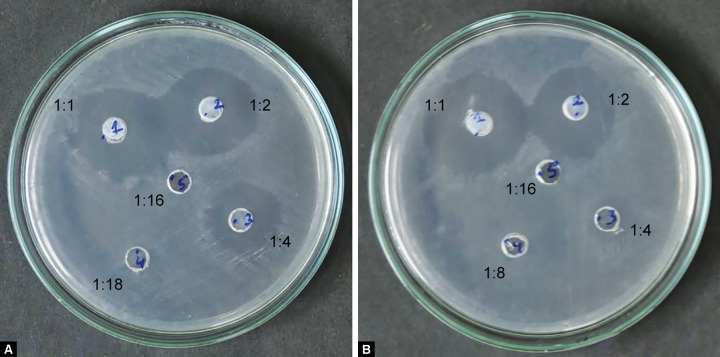
Inhibitory halo for samples D and E (0.35% MFP) at 1:1, 1:2, 1:4, 1:8, and 1:16 dilutions

**Fig. 4:**
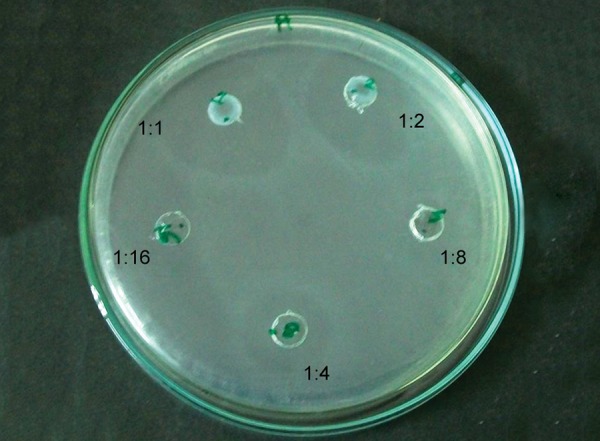
Inhibitory halo for sample F at 1:1, 1:2, 1:4, 1:8, and 1:16 dilutions

**Fig. 5:**
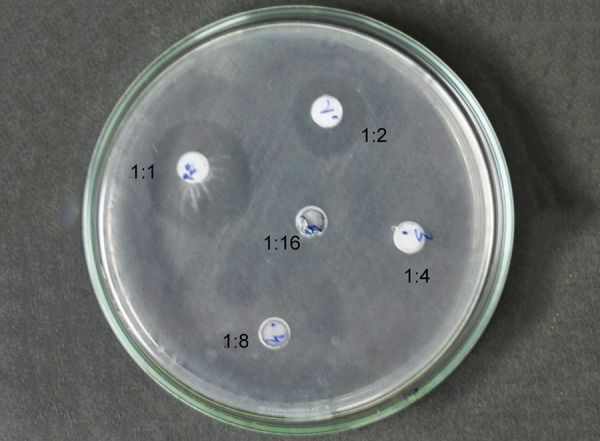
Inhibitory halo for sample G at 1:1, 1:2, 1:4, 1:8, and 1:16 dilutions

Figures 1 to 5 show the inhibitory halos for samples A to G at 1:1, 1:2, 1:4, 1:8, and 1:16 dilution. Sample E showed the maximum zone of inhibition (29.17 ± 0.764 mm), while sample C showed the weakest activity (8.73 ± 0.115 mm) at 1:1 dilution. Sample F showed an effective result till the 1:8 dilution (10.97 ± 0.252). No inhibitory circles were seen at 1:16 dilution (Table 1).

## DISCUSSION

*In vitro* studies have demonstrated that *S. mutans* produces less acid when a low concentration of fluoride is constantly present. Fluoride concentrates in dental plaque and inhibits metabolizes of carbohydrate (by cariogenic bacteria), leading to lesser production of lactic acid. It also affects the bacterial production of adhesive polysaccharides.^6^

This study examined the antimicrobial effect of pediatric dental formulations containing NaF, sodium MFP - 0.35% and 0.38% concentration, AmF, and calcium phosphate utilizing laboratory strains of *S. mutans* bacteria. Due to the risk of fluorosis^7^ and other systemic effects associated with fluoride, pediatric toothpaste have almost half the concentration of fluoride than in adult dental formulations. Despite the lower fluoride concentration, the pediatric dental formulas are proved to be antimicrobial against the tested strain. Out of these, sample E containing 0.35% MFP showed maximum zones of inhibition (29.17 ± 0.764) and sample C containing 0.38% MFP showed the lowest inhibitory circle (8.73 ± 0.115; Table 1, Figs 2 and 3). The result in the present evaluation is in good agreement with previous systemic review that efficacy of a fluoridated toothpastes in anticariogenic properties is better than other nonfluori-dated toothpaste.^8^

Among the different fluoride combinations, sodium MFP (sample E) was found to be more effective than NaF (sample A) and AmF (sample F). As a topical agent, MFP is an effective caries inhibitor^9^ and thus it is the most commonly used active ingredient dental formulation. In this form, the fluoride is tightly covalently bounded and requires enzymic hydrolysis to release fluoride ions.^10^ Thus, more amount of free active fluoride is available over the tooth surface, unlike NaF combinations that react with the filler particles, thus reducing amount of active fluoride available.^10^

Reed^11^ showed clinical effectiveness of toothpaste was proportional to total fluoride concentration when using NaF. However, this proportionality does not hold for MFP toothpaste^9^; 0.35% MFP (samples D and E) was found to be more effective than 0.38% MFP (samples B and C) toothpaste. A less than expected response of 0.38% MFP (samples B and C) was seen. This may be due to chemical interaction with other ingredients, which renders fluoride unavailable on the tooth surface.^12^ Thus, it can be interpreted that the uptake of free fluoride ion for MFP does not depend upon the total concentration of fluoride present.

To verify the altered response of sodium MFP, we used different toothpastes with same MFP concentrations (0.35 and 0.38%) and different sugar substitute. So, with each sodium MFP formulation, xylitol and sorbitol were also considered. There seem to be an interaction with sugar substitute with 0.35% MFP. But the results were found to be almost the same with 0.38% MFP.

The mean value of inhibitory circles for sample D (0.35% MFP and xylitol sugar substitute) at 1:1 and 1:2 dilutions was found to be almost equal (Fig. 3). This may be due to presence of an effective 0.35% MFP along with the polyhydroxy compound - xylitol as a sugar substitute. Xylitol in dentifrice reduces both dental plaque composition and salivary levels of mutans streptococci.^13^

A prolonged and an effective response of AmF formulation was seen (Fig. 4). This may be attributed to the fact that amine residues of AmF also possess antibacterial properties due to its positively charged amine part.^14^ Schiller et al^15^ have demonstrated an effective antimicrobial action of Elmex® against *S. mutans.* Amine fluoride when used in combination with stannous fluoride shows an outstanding antimicrobial effect, inhibits plaque formation, and prevents inflammation.^16^ No such toothpaste formulation is available for kids; however, Meridol^®^ toothpaste is a commercially available combination of AmF and stannous fluoride.

## CONCLUSION

Based on the data obtained from the present study, it can be concluded that:

- In spite of lower fluoride concentration, kids dental formulas show antimicrobial activity against *S. mutans in vitro.*
- Further dilutions in the dentifrices can be made possible, thus lowering the fluoride concentration in kids dentifrices with a considerably good amount of antimicrobial activity.
